# “*Question Your Categories”*: the Misunderstood Complexity of Middle-Distance Running Profiles With Implications for Research Methods and Application

**DOI:** 10.3389/fspor.2019.00028

**Published:** 2019-09-26

**Authors:** Gareth N. Sandford, Trent Stellingwerff

**Affiliations:** ^1^School of Kinesiology, University of British Columbia, Vancouver, BC, Canada; ^2^Physiology, Canadian Sport Institute-Pacific, Victoria, BC, Canada; ^3^Athletics Canada, Ottawa, ON, Canada

**Keywords:** anaerobic speed reserve, training science, fiber type, individualized training, coaching, bioenergetics

## Abstract

Middle-distance running provides unique complexity where very different physiological and structural/mechanical profiles may achieve similar elite performances. Training and improving the key determinants of performance and applying interventions to athletes within the middle-distance event group are probably much more divergent than many practitioners and researchers appreciate. The addition of maximal sprint speed and other anaerobic and biomechanical based parameters, alongside more commonly captured aerobic characteristics, shows promise to enhance our understanding and analysis within the complexities of middle-distance sport science. For coaches, athlete diversity presents daily training programming challenges in order to best individualize a given stimulus according to the athletes profile and avoid “non-responder” outcomes. It is from this decision making part of the coaching process, that we target this mini-review. First we ask researchers to “question their categories” concerning middle-distance event groupings. Historically broad classifications have been used [from 800 m (~1.5 min) all the way to 5,000 m (~13–15 min)]. Here within we show compelling rationale from physiological and event demand perspectives for narrowing middle-distance to 800 and 1,500 m alone (1.5–5 min duration), considering the diversity of bioenergetics and mechanical constraints within these events. Additionally, we provide elite athlete data showing the large diversity of 800 and 1,500 m athlete profiles, a critical element that is often overlooked in middle-distance research design. Finally, we offer practical recommendations on how researchers, practitioners, and coaches can advance training study designs, scientific interventions, and analysis on middle-distance athletes/participants to provide information for individualized decision making trackside and more favorable and informative study outcomes.

## Introduction

In the book *Factfulness* the late Professor Hans Rosling addresses “Ten reasons why we're wrong about the world” (Rosling et al., 2018). Specifically, Rosling explains how we subconsciously employ bias in our decision making and interpretation of the world based on self-narrative, which can drive false positive and negative understanding of reality. Accordingly, we will apply one of Professor Rosling's ten-principles: “*Question your categories”* to an approach generally employed by many middle-distance researchers; in that, generally, many take a singular approach to the treatment and analysis of middle-distance athletes and/or study participants. Biological first principles consistently demonstrate a huge variability in adaptation to a given exercise stimulus and is prevalent across multiple sports (Gaskill et al., 1999; Vollaard et al., 2009; Timmons et al., 2010; Sylta et al., 2016). Therefore, a “one-size-fits-all” approach needs re-consideration based on substantial individual responses to a given stimulus, that is especially unique to the middle-distance event group resulting in very different physiological and mechanical profiles achieving similar elite performances (Schumacher and Mueller, 2002; Sandford et al., 2019a). Many in the middle-distance coaching community already generally implement individualized training (Horwill, 1980; Daniels, 2005), but highlight the need for deeper information surrounding how to best address the complexity of middle-distance athletes. It is from this coaching perspective that we target this mini-review, providing recommendations on how researchers/practitioners can advance training study designs, scientific interventions, and analysis in middle-distance athlete profiles research to provide more beneficial information for individualized decision making and/or more favorable and informative study outcomes.

## What Constitutes Middle-Distance?

Consistency of both sport science terminology (Chamari and Padulo, 2015; Winter et al., 2016) and grouping of middle-distance events and athletes within the literature is lacking. Therefore, initially, we put forward a framework for defining middle-distance running events as *solely* the 800 and 1,500 m events (~1.5 to 5 min duration; Table 1); primarily due to the demarcation of average 800 and 1,500 m race pace intensity in relation to a given physiological threshold (beyond VO_2_max; Table 1).

**Table 1:** Proposed framework for standardizing researcher and practitioner categories of events 800 m—marathon considering both average race velocity and subsequent physiological consequences of a given race demand.

**Parameter**	**Middle-distance**		**Middle-long distance**			**Long distance**	
**Events**	**800 m (min:ss:ms)**	**1,500 m (min:ss:ms)**	**3,000 m (min:ss:ms)**	**5,000 m (min:ss:ms)**	**10, 000 m (min:ss:ms)**	**60 min record (^**~**^l/2 marathon) (min:ss)**	**Marathon (hr:min:ss)**
Male world record event duration (hr:min:ss:ms)	1:40.91	3:26.00	7:20.67	12:37.35	26:17.53	58.18	2:01:39
Average race pace intensity (% VO_2_max; Billat, 2001)	115–130	105–115	^~^100	95–100	90–95	85–90	75–80
Physiological threshold	Above VO_2_max		≤VO_2_max, ≥ Critical velocity			<Critical velocity	
% Aerobic energy contribution (Billat, 2001)	65–75	80–85	85–90	90–95	97	98	99.9
% Aerobic energy contribution (Spencer and Gastin, 2001)	66 ± 4	84 ± 3	n/a	n/a	n/a	n/a	n/a
% Aerobic energy contribution (Duffield et al., 2005a,b)	60.3 ± 9	77 ± 7	86 ± 7	n/a	n/a	n/a	n/a
Coach interpretation of % aerobic energy contribution (Gamboa et al., 1996)	35–65	n/a	n/a	n/a	n/a	n/a	n/a
% difference in aerobic contribution to 800 m	–	5–20	10–25	20–30	22–32	23–33	24.9–34.9

*Adapted from Gamboa et al. (1996); Billat (2001); Spencer and Gastin (2001); Duffield and Dawson (2003); Duffield et al. (2005a,b)*.

The distinction of middle-distance as solely 800 and 1,500 m is critical for advancing current understanding. First, between 0 and 5 min, performance decrements of all-out efforts are exponential as a function of time (Bundle and Weyand, 2012). Therefore, within this time frame, a varying blend, but still large contributions, of (1) aerobic, (2) anaerobic, and (3) neuromuscular/mechanical characteristics are implemented to achieve optimal performance (Schumacher and Mueller, 2002; Sandford et al., 2019a). An appreciation of the differences in these three distinct performance determinants between, and within, middle-distance events has received limited consideration within the middle-distance literature, largely perhaps due to our limitations in accurately and reliably quantifying anaerobic energetics (Haugen et al., 2018). If one extends the middle-distance category beyond ~5 min (for example to 7,8, or 15 min) a much smaller decline in performance is seen (between e.g., 5 and 9 min than between 1 and 5), due to the similar nature of aerobic contribution support the extended duration (e.g., 5–9 min) of exercise (Bundle and Weyand, 2012; Table 1). Second, the average race pace of 800–1,500 m as a % VO_2_max, are beyond VO_2_max, providing distinctly different metabolic consequences to those events that are below VO_2_max, both of which are distinctly different to those events that reside, on average, below critical velocity (defined as the last wholly oxidative physiological intensity; Table 1). Therefore, it is important that in establishing middle-distance event specific performance determinants, and/or appropriate performance enhancing interventions, that the bioenergetics and neuromuscular/mechanical requirements represent the actual demands from ~1.5 to 5 min of duration, which are much different if one includes middle to long and long distance athletes (Table 1).

Consequently, training and applying interventions to athletes within this middle-distance event group are much more divergent than many practitioners and researchers appreciate. Indeed, recent work in elite 800 m runners has shown huge diversity of profiles presenting along the continuum of middle-distance running (Sandford et al., 2019a), which we will discuss below. Accordingly, we suggest that given the unique bioenergetics (Table 1) and neuromuscular/mechanical constraints, that middle-distance are exclusively defined as the 800 and 1,500 m athletics events, or ~1.5 to 5 min of duration.

## Middle-Distance Running—The Event Group With Largest Diversity of Athlete Profile?

**Figure 1:**
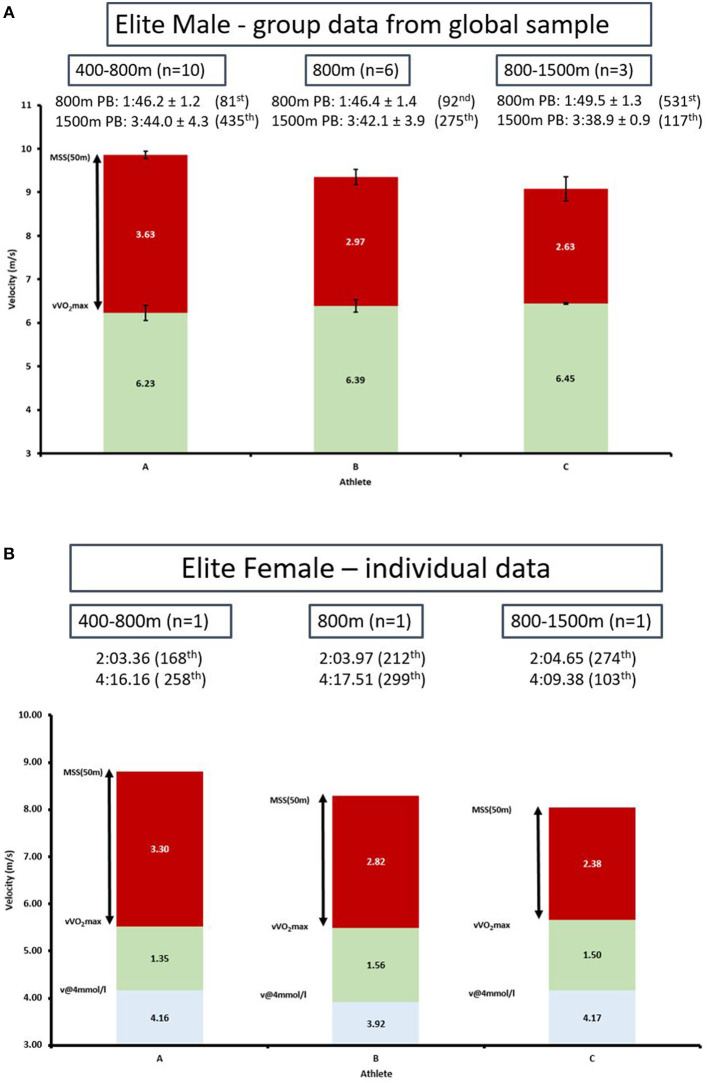
**(A)** Anaerobic speed reserve profiles of 19 elite male 800 and 1,500 m athletes across 800 m sub-group continuum as described in Sandford et al. (2019a). All participants seasons best (SB) 800 m ≤1:47.50 and 1,500 m SB ≤3:40.00. vVO_2_max estimated from 1,500 m race time as per methods of Bellenger et al. (2015) and validated in elite male runners in Sandford et al. (2019b). **(B)** Anaerobic speed reserve and velocity at 4 mmol/l lactate (v@4 mmol/l) across three elite middle-distance female profiles from each of the 800 m sub-groups tested in 2017. Note the between individual diversity across v@4 mmol/l, vVO_2_max and Maximal sprint speed—despite all having a season's best over 800 m within 1.3 s of each other. Rankings in brackets from 2017 season. vVO_2_max generated using methods developed by Bellenger et al. (2015) and utilized in Sandford et al. (2019a) **(A)**. Informed consent was obtained through Auckland University of Technology ethics committee as part of Sandford et al. (2019a).

The middle-distance events are described as the “middle-ground” of aerobic and anaerobic energetics (Billat, 2001; Table 1), where, accordingly, athletes may approach the same performance time from distinctly different perspectives, as shown by diversity of aerobic energetics within the 800 m (Table 1). Indeed, most coaches appreciate the large variability of aerobic energetics across the 800 m event when programming training (Gamboa et al., 1996), which actually aligns well with the published diversity of energetic contributions (Table 1). As an example, published case studies on world-class 1,500 m runners Henrik Ingebrigtsen (Tjelta, 2013) and Peter Snell (Carter et al., 1967) show substantial diversity in physiology (VO_2_max) and performance profile at 800 and 3,000 m, despite similar 1,500 m race performances (3:35.43 and 3:37.60, respectively, at time of publication). For example Ingebrigtsen presents with a VO_2_max of 84.4 vs. Snell's value of 72.2 ml/kg/min. Ingebrigtsen's personal best at 800 and 3,000 m are 1:48.60 and 7:58.15, respectively. By comparison, over the 800 m Snell recorded 1:44.30 world-record and had no recorded 3,000 m race performances (but did record 9:16 on grass and 9:12.5 on cinder tracks over 2 miles (*Steve Willis personal communication*), which converts to 8:36.10 and 8:32.90 3,000 m (IAAF scoring tables). Therefore, despite these two athletes having similar 1,500 m race times, it is obvious that Snell's 1,500 m performance comes much more from a speed/anaerobic physiological profile compared to the more aerobic profile from Ingebrigtsen. In further support of divergent middle-distance athlete profiles, a recent global sample by Sandford et al. (2019a) revealed substantial diversity of athlete profile within elite male 800 m athletes, that can be categorized by three distinct sub-groups (400–800 m speed types, 800 m specialists and 800–1,500 m endurance types) across a continuum (Figure 1A). The same within 800 m subgroups are also found in elite females (Figure 1B; where velocity at 4 mmol (v4mmol) has also been added). Providing just three measures of an athletes profile, such as v4mmol (aerobic indicator), velocity at VO_2_max (vVO_2_max; aerobic power indicator) and maximal sprinting speed (MSS; biomechanical/structural indicator, measured over 50 m from standing start, Sandford et al., 2019a) provides a great “first layer insight” for any researcher, practitioner, and coach. From this, one can more easily identify: (a) the physiological and biomechanical strengths and limitations of an individual; and (b) which “sub-group” the athlete/participant is currently in. This, in turn, potentially enables more targeted interventions by sub-group to improve depth of understanding on stimulus-response of interventions across the middle-distance continuum which ultimately aid the ability to inform individualized training prescription.

It is important to note, that without the characterization of MSS in these middle-distance athletes, something which is rarely reported in middle-distance studies, this continuum characterization is not possible (Figures 1A,B). Interestingly, these identified middle-distance athlete sub-groups (Sandford et al., 2019a) supports longstanding coaching observations of middle-distance athlete variability that requires careful individual considerations (Horwill, 1980).

We suggest that appreciating the continuum of middle-distance diversity is currently poorly implemented in many research study designs, and poorly appreciated amongst middle-distance researchers and practitioners. Accordingly, as outlined below and in Table 2, considerations of this diversity should occur with: (l) section Study Athlete/Participant Characterization and Description (II) section Selection of Appropriate Intervention - Are All Stimulus Created Equal?; and (III) section Analysis of Effects per Sub-group.

**Table 2:** Study design principles for middle-distance running populations.

**Example Research Question: “The effect of high intensity training interventions at or beyond VO**_****2****_**max on middle-distance race performance”**				
	**Traditional approach**	**Issues with approach**	**Emerging approaches**	**Rationale**
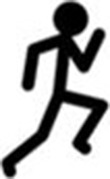 I. Participant description	“18 middle-distance runners,” height, weight, age, international ranking level, VO_2_max, middle-distance performance times	Does not provide enough information to distinguish what type of middle-distance athlete the participants since MSS is not assessed	MSS measured over 50 m used to complement the aerobic characterization of vVO_2_max. Elite Athletes presented across the middle distance continuum, *n* = 400–800, *n =* 800, and *n* = 800–1,500 and categorized using the SRR (MSS/vVO_2_max) (Sandford et al., 2019a) Performance times provided across multiple distances (400, 800, 1,500) Training volume, time in zones quantified to understand training history	Allows for sub-group characterization even in underpowered studies that can have closer application and relevance for coaches and support staff frontline or for future study hypothesis generation
II. Exercise prescription	%vVO_2_max (>VO_2_max) %HRmax (>VO_2_max) Event personal best running speeds (e.g., intervals at 800 or 1,500 m race pace)	Human locomotor performance (i.e., time to exhaustion) at intensities beyond vVO_2_max can be surprisingly “predicted” using only 2 locomotor entities: vVO_2_max and MSS (Bundle et al., 2003; Alexander, 2006; Bundle and Weyand, 2012) Therefore, without acknowledgment of the MSS differences, the intervention will have athletes working at different relative intensities of a given workload. For example, the lower the use of the ASR, the greater the exercise tolerance (Blondel et al., 2001; Buchheit et al., 2012; Buchheit and Laursen, 2013) and thus a big confounder often overlooked in these types of studies	% ASR (or exercise prescription decisions set relative to both %vVO_2_max and %MSS)	Accounts for mechanical differences between athletes and allows the same relative physiological stimulus to be applied (Buchheit and Laursen, 2013)
Ill. Analysis	(1) All runners grouped together for analysis (despite some studies having 800 m (1.5–2 min) to marathon (130–160 min) specialists. (2) Periodically a sub-group responders vs. non responders	Misrepresentation of athletes ability to “respond.” Should we expect all athletes to respond equally to the same stimulus despite having very different event specialty or diverse profile to approach the same event?	Analyze data as a single group, BUT also display individual and sub-group response and differences between subgroups	Further understanding the appropriateness of a stimulus for a given sub-group profile

### Study Athlete/Participant Characterization and Description

All studies are required to profile and characterize their participants (Begg et al., 1996). Typically, many middle-distance based studies tend to limit this reporting to primarily aerobic based physiological parameters, such as VO_2_max (or vVO_2_max), lactate threshold, and performance times and participant age and anthropometrics. Sometimes, depending on the scope of the study, some anaerobic metrics are provided, appreciating sport science currently has limited validity in accurately and sensitively measuring the anaerobic domain (Haugen et al., 2018). Furthermore, middle-distance coaching education is predominated by aerobic based energy system teaching (Berg, 2003; Thompson, 2016; Sandford, 2018), which may skew the over-emphasis on these performance elements.

In Rosling's words we should look to “get-a tool box not a hammer.” Accordingly, we suggest that neuromuscular and mechanical qualities, such as MSS and the anaerobic speed reserve (ASR, defined as the speed range from Velocity at VO_2_max to MSS, Blondel et al., 2001; Buchheit and Laursen, 2013), offer potential to deepen our understanding of athlete profile diversity. At the very least, the addition of MSS allows for enhanced potential analysis (see Figures 1A,B), and is a technically and methodologically easy addition to most study designs. However, very rarely, are any maximal speed/power and/or biomechanics based metrics reported or considered (despite considerable evidence showing them to be important performance determinants of MSS Weyand et al., 2000, 2010; Morin et al., 2011; Rabita et al., 2015; Nagahara et al., 2019) as well as determinants of middle-distance race performance (Nummela et al., 1996; Bachero-Mena et al., 2017; Sandford et al., 2019a).

Furthermore, many papers only report single event performance, which does not inform the reader on where the strengths and weaknesses of a given athletes/participants lie (e.g., Figures 1A,B). Athlete/participant profiles may be further characterized by concepts such as ASR and the speed reserve ratio (SRR; MSS/vVO_2_max) allowing authors to describe the distribution of their athlete/participants sub-group(s). As a minimum authors should show a spread of performance times, for example 400, 800, and 1,500 m personal bests. Expanding upon the athlete/participant profile in a study design allows for significant improvement in analysis as well as for applied sport practitioners and coaches to determine the relevance of study findings to the athletes they coach.

### Selection of Appropriate Intervention—Are All Stimulus Created Equal?

Many papers report responder or-non-responder outcomes following a blanket intervention without inspection of participant profile diversity (see: Gaskill et al., 1999; Vollaard et al., 2009; Timmons et al., 2010; Sylta et al., 2016). This may result in assuming a “non-response.” Conversely, perhaps an inappropriate stimulus was implemented for their unique sub-group profile that has created the “non-responder” outcome, rather than the athlete's inability to adapt. Equally, the same stimulus may have favored other uniquely identified subgroups in the sample resulting in responders. Such scenarios are daily challenges in coaching and an area where furthering our scientific approach could add great resolution to inform frontline decision making. Interestingly, a recent paper highlighting the value of an individualized training intervention, albeit in a team sport group by Jiménez-Reyes et al. (2017) demonstrate that individualized programming based on a subjects baseline force-velocity profile led to greater improvements in jump performance, with less variability, compared to a generic non-individualized strength training programme.

One major mechanism (but not exclusive from other neural and morphological components) underpinning the diversity of middle-distance athletes and unique adaptive profiles might be muscle fiber typing. Slow twitch muscle are characterized by myosin heavy chains (MHC) I and fast twitch by MHC II [sum total of MHC lla (fast oxidative) and IIx (fast glycolytic)], and shall be discussed using these isoforms herein. Historical understanding of fiber typing at the extremes of speed and endurance have been well-understood since the 1970s (Costill et al., 1976), but the blend of these qualities in the middle-distance are less clear (van der Zwaard et al., 2017). MHC IIa and IIx fiber composition is a common characteristic underpinning elite speed and power performance. For instance, a former world champion sprint hurdler demonstrated an impressive 71% MHC II (24%llx) (Trappe et al., 2015). MHC II also has a superior ability to hypertrophy (Billeter et al., 2003). Further characteristics of MHC II muscle include larger baseline muscle carnosine content (Parkhouse et al., 1985; Baguet et al., 2011) that have been related to frequency of movement (i.e., more MHC II, higher frequency of movement) (Bex et al., 2017); and enhanced muscle buffering. In addition, greater creatine content is also found at rest in MHC II muscle, which supports more anaerobic based exercise (Tesch et al., 1989). All of these facets have implications for muscle buffering capacity, sensitivity to supplementation and intervention designs with ergogenic aids (Stellingwerff et al., 2019).

By contrast distance runners (5 km–to marathon) have shown a MHC I fiber range of 63.4 to 73.8%, with 1972 Olympic Marathon gold-medalist Frank Shorter having 80% MHC I (Costill et al., 1976). Taken together, differences in fiber typing and specific hypertrophy, highlights the complexity of any one type of stimulus to a phenotypic adaptive outcomes that requires careful future sub-group investigation.

In the middle of this MHC I-to-MHC II continuum lie the middle-distance athletes (Costill et al., 1976; Baguet et al., 2011). The concurrent event demand for middle-distance athletes of speed and endurance is at conflict with the inverse relationship between oxidative capacity and muscle cross sectional area (CSA—where MHC II are larger), alongside the strong relationship between MHC I and oxidative enzyme activity (Zierath and Hawley, 2004; van der Zwaard et al., 2016). Therefore, a given middle-distance athlete may present from varying points along this fiber-type continuum. For example, Costill et al. (1976) revealed a large MHC I range of 44.0–73.3% and 40.5–69.4% in female and male middle-distance runners, respectively. Interestingly, these fiber type ranges overlaps with the aerobic contribution to the 800 and 1,500 m events (Table 1).

Without separating the presenting diversity into sub-groups in our study designs, we are potentially blurring the individuality of responses that may be present, and thus, perhaps losing effects that work for some sub-groups and not others. An alternative may be to consider what interventions may be appropriate for a given sub-group within a study population, rather than applying a generic intervention to all participants; much like a coach does daily in prescribing training for their athletes.

### Analysis of Effects per Sub-group

The consequence of employing blanket interventions to one group is the “signal” of the effect may be lost in the diversity of the athlete sample, which presents as non-significant “noise.” Therefore, approaches such as ASR, alongside measures of critical velocity/v4mmol/l, can allow for significantly enhanced data analysis. In the end, it is best to not choose one model, but a broad perspective (multidisciplinary approach) to fully develop the athlete profile and subsequent analysis. In addition consideration of mechanical differences such as aerial or terrestrial profiles (Lussiana and Gindre, 2016) or baseline muscle carnosine (Baguet et al., 2011), representative of fiber-typing could add huge value in characterizing and determining effective interventions for the different sub-groups. Given some research interventions will have more relevant categories than others (e.g., aerial vs. terrestrial biomechanics vs. ASR sub-group using SRR vs. baseline muscle carnosine/creatine), consider presenting results using multiple layered sub-groups (e.g., 400–800 m athlete aerial profile vs. 400–800 m terrestrial profile), to potentially provide a more complete understanding of the complex characteristics between and within middle-distance athletes. Finally, the smallest worthwhile change to competitive performance in elite—middle-distance running (defined as <3 km) is 0.5% (Hopkins, 2005). Bringing to question whether our investigations and groupings of 800–5,000 m as “middle-distance,” with up to 30% difference in aerobic energetic demands (Table 1) are too broad to determine an effect that matters to performance within the sub-group complexity.

## Conclusion and Recommendations

In the present mini-review we, first, provide a call to action for authors to “*Question your categories”* with regards to broad unidimensional classification of the middle-distance running events. Second, we outline multiple areas at an athlete/participant level where research design and consideration for sub-group outcomes at multiple steps (section Study Athlete/Participant Characterization and Description, Selection of Appropriate Intervention—Are All Stimulus Created Equal?, Analysis of Effects Per Sub-group; Table 2) can enhance the application of research to the coach and practitioner frontline. Until the inherent diversity of athlete profiles are appreciated by the middle-distance research and practitioner community, many current generic middle-distance sport science recommendations and associated research methods will continue to provide a misleading narrative and understanding of effective middle-distance interventions. It is for sport scientists at the frontline to connect the sub-group understanding and characterization from the lab to the track, enabling our coaches to make the most informed recommendations about individualizing interventions based on the athlete presenting in front of them.

To conclude, in the words of Professor Hans Rosling “*It will be helpful to you if you always assume your categories are misleading. Here are five powerful ways to keep questioning your favorite categories: look for differences within and similarities across groups; beware of the majority; beware of exceptional examples; assume you are not normal; and beware of generalizing from one group to another.”*

It is from this paradigm that we believe more progress will be made in understanding the complexities, and training stimulus approaches in applied sport science application to middle-distance running.

## Author Contributions

GS and TS were involved in the conceptual ideas, writing a first draft of the paper, selection and production of figures and tables, and revising the manuscript.

### Conflict of Interest

The authors declare that the research was conducted in the absence of any commercial or financial relationships that could be construed as a potential conflict of interest.
